# Contributions to the Medical Press

**Published:** 1873-07

**Authors:** 


### EDITORIAL AND MISCELLANEOUS.

### CONTRIBUTIONS TO THE MEDICAL PRESS.

It has doubtless been observed by our readers, with satisfaction, that the recent numbers of this journal have been made up in very large part of original matter.

Why should not this feature be permanently continued? We firmly believe that it is both the duty and the interest of *every one* of our readers to become, likewise, a contributor to our columns. We think this belief is susceptible of demonstration.

The fund of medical lore, be this small or great, with which we leave the lecture benches and the hospital, to engage actively in the contest with disease, is drawn from a common fund, which embodies the small individual contributions of medical men in all ages of the world. From this great store-house of knowledge we are permitted to make our drafts at will, and this without money and without price. But while we are thus free to imbibe knowledge from a common fountain, in doing so we place ourselves under a moral obligation to so use the information imparted to us as that it shall in turn feed the little streams that keep the fountain ever full to overflowing. He who, having received at the hands of the masters a talent, however small, buries that talent in the ground, falls short of the discharge of a plain, manifest duty.

Moreover the interests, both professional and pecuniary interests, of the physician, are materially subserved by his contributions to medieal science. In this it may truthfully be said, more blessed is he who gives than he who receives. It may not be unprofitable to examine briefly some of the advantages which are to be gained by contributors to the medical press.

The practice of putting one's thoughts upon paper, and especially for the medical press, engenders habits of systematic thought and reasoning.

It stimulates research, and adds little by little, but nevertheless surely, and in the aggregate surprisingly, to the writer's fund of medical knowledge.

It heightens the interest with which the contributions of others are read.

It extends materially and most advantageously the sphere of one's professional fame and influence.

It cultivates relations of professional esteem and friendship far and wide amongst those who are beyond the reach of personal intercourse.

It stamps those who practice it as *live men* in the esteem of their fellows and in the estimation of the world.

It stores away in an enduring form the fresh thought and observation of *to-day*, where it will be readily accessible for use in after years,—thought and observation, often, whose value the future alone can develop, and which, if entrusted to the keeping of treacherous memory, is necessarily impaired, and, too frequently, entirely lost.

It affords one a valuable opportunity of measuring himself intellectually and professionally with his fellows. It discovers his weak points that he may exert himself to strengthen them; and likewise his strong points, that he may take comfort therefrom and press more bravely onward.

*In our profession, to be widely and at the same time favorably known is fame, and fame is money.*

We respectfully urge upon our readers the duty and the advantage of becoming contributors to our columns. Let them note down their interesting cases and their individual observations and put the information upon record for their own future use, and for the common use of the brethren.

To any who may desire it, we cheerfully proffer our editorial aid in preparing their notes and observations for presentation to the public. Furnish us with the facts, and we will gladly put them into form to be acceptable to our readers. B.

---

ADDRESS all communications regarding in any way the business department of the JOURNAL—viz: those containing money and post-office orders, or relating to advertisements, subscriptions, or failures to receive the JOURNAL—to the publishers, "Herald Publishing Company."

